# Psychosocial challenges of persons with sickle cell anemia: A narrative review

**DOI:** 10.1097/MD.0000000000036147

**Published:** 2023-11-24

**Authors:** Emmanuel Aniekan Essien, Blessing F. Winter-Eteng, Chinyere Uchechi Onukogu, Dominic Dennis Nkangha, Faithful Miebaka Daniel

**Affiliations:** a Research and Training Unit, Federal Neuropsychiatric Hospital; b Community and Clinical Research Division, First On-Call Initiative.

## Abstract

Sickle cell anemia (SCA) is a severe form of sickle cell disease that primarily affects black populations and individuals in tropical countries. This condition causes significant morbidity and mortality and leads to a range of psychosocial challenges. A preliminary search was conducted on Ovid Medline and public databases with a combination of Medical Subject Headings keywords, resulting in 368 articles. The articles were screened based on the selection criteria in a nonsystematic method by 3 researchers, and a narrative synthesis was done to analyze extracted data from selected peer-reviewed article. Mental disorders, sleep disturbances, interpersonal relationship challenges, stigmatization, and workplace discrimination were identified as significant contributors to the psychosocial distress experienced by individuals with SCA and their families. Depression and anxiety were prevalent among individuals with SCA, leading to poor treatment adherence, increased pain, and disruptions in various aspects of life. Sleep disturbances, including sleep-disordered breathing and sleepwalking, were also identified as significant contributors to poor sleep quality in SCA patients. Families of individuals with SCA also face challenges, including psychological stress, financial strain, and social disruption. Stigmatization is common, leading to misconceptions and discrimination. Workplace discrimination is prevalent, with a high unemployment rate among adult SCA patients. Comprehensive care is crucial to address these psychosocial issues. Early identification and intervention, comprehensive support programs, patient and family education, enhanced pain management strategies, and integration of mental health into clinical care are recommended. School-based support, research and advocacy, and community support groups are also important. By addressing these challenges through comprehensive care and support, healthcare professionals, policymakers, and society can reduce psychosocial distress and improve the lives of individuals with SCA.

## 1. Introduction

Sickle cell disease (SCD) is a group of hematological disorders characterized by abnormal hemoglobin, leading to defective red blood cell morphology and function.^[1]^ The most severe variant of SCD is sickle cell anemia (SCA), caused by an autosomal recessive mutation in the β-globin hemoglobin gene.^[1]^ The disease has an uneven distribution documented in literature, and much is known about the prevalence of the disease. However, poor early neonatal screening for hemoglobin genotype hampers reliance on data in resource-poor settings. This global disease affects approximately 300,000 babies annually, with 2-thirds of cases occurring in Africa.^[1]^ Tropical countries like Nigeria, India, and the Democratic Republic of Congo bear the highest burden of SCA.^[1]^ The pathological consequences of SCA are extensive and affect various aspects of an individual health and have continued to be the subject of research interest. The hallmark of SCA is the abnormal sickling and loss of pliability of red blood cells, leading to vaso-occlusive crises (VOC), hemolytic crises, chronic anemia, and severe pain episodes.^[2]^ Additionally, individuals with SCA are prone to strokes, recurrent infections, avascular necrosis, priapism, and growth delay.^[3]^ While advancements in medical interventions have improved life expectancy for SCA patients in high-income countries, the situation remains challenging in low-resource settings.^[4]^ The lack of healthcare infrastructure and policies in these regions contributes to poorer outcomes for individuals with SCA.^[4]^

In addition to the medical syndromes experienced by individuals with SCA, there is an interplay of psychosocial challenges, including mental disorders, pain crises, sleep disorders, interpersonal relationships, substance abuse, stigma, and workplace discrimination, as suggested by literature.^[5–10]^ However, a wider array of psychosocial challenges impacting the patients, their family and peers, and ignored can affect overall response to management, has been underrepresented in literature.^[11]^ As a result, SCA patients have reported negative experiences due to practitioners ignoring or undermining the effect of these less overt challenges. Such experiences include stigma produced discrediting pain reports, labeling and stereotyping, blaming patients for not improving their health, discrimination, racism, inadequate pain assessment, and delay in care.^[12]^ On the other hand, there is evidence on the benefits of social support and programs that lead to overall improvement in health-related quality of life (QOL) and coping.^[13–15]^ However, these programs only exist where there is adequate knowledge of the problem and the necessity. Therefore, it is pertinent to review this subject at a global scale to provide an elaborate perspective of the problem and what policies or intervention strategies have proven to be effective and can be domesticated in other climes. This review aims to summarize findings on the prevalence and impact of mental disorders such as anxiety and depression, pain experiences, interpersonal relationships, workplace discrimination, stigma, sleep disorders, and body dissatisfaction among individuals with SCA. These are important factors to consider in addressing psychosocial challenges and improving management strategies. By recognizing and addressing these issues, healthcare providers can prioritize holistic care for SCA patients and improve the overall management and outcomes for individuals living with SCA.

## 2. Methodology

To address the aims of this review, we attempted answer the following reviewing questions: How prevalent are mental disorders such as anxiety and depression among individuals with SCA, and what is the impact of these disorders on coping mechanisms, interpersonal relationships with peers and family, and overall psychosocial well-being? What is the prevalence and impact of workplace discrimination, stigma, sleep disorders, and body satisfaction among individuals with SCA, and how do these factors influence mental health, substance use, pain experiences, and overall psychosocial challenges?

### 2.1. Search strategy

A literature search was conducted to identify relevant articles from electronic databases such as PubMed, Embase, PsycINFO, and Scopus. The search strategy included a combination of Medical Subject Headings keywords on Ovid Medline using the following keywords: “anemia, sickle cell,” “mental health,” “social stigma,” “social support,” “quality of life,” “adaptation, psychological,” “mental disorders,” “sickle cell anemia AND mental disorders,” “sleep,” “substance-related disorders,” “pain,” “family relations,” “body dissatisfaction,” “interpersonal relations,” “psychology,” “pain AND psychology,” “medical psychology,” “social stigma OR social support OR quality of life OR sleep OR substance-related disorders OR Family relations OR body dissatisfaction OR interpersonal relations OR (pain AND psychology),” “sickle cell anemia AND social stigma OR social support OR quality of life OR sleep OR substance-related disorders OR Family relations OR body dissatisfaction OR interpersonal relations OR (pain AND psychology),” “social stigma OR social support or psychological adaptation or substance-related disorders or family relations or interpersonal relations or (pain AND psychology),” “sickle cell anemia AND [social stigma OR social support or psychological adaptation or substance-related disorders or family relations or interpersonal relations or (pain AND psychology)].”

### 2.2. Study selection criteria

Studies were included if they met the following criteria: were peer-reviewed articles, focused on psychosocial issues in SCA, included participants diagnosed with SCA of any age group, published in the English language, and published between 1989 and 2023. Quantitative and qualitative studies were considered for inclusion, including cross-sectional, longitudinal, and intervention studies (Table 1).

**Table 1:** Showing article selection process for reviewing psychosocial issues and sickle cell anemia (SCA).

Stage	Description	Number of articles
Initial search	Database search using keywords like “anemia, sickle cell,” “mental health,” “social stigma,” “social support,” “quality of life,” “adaptation, psychological,” “mental disorders,” “sickle cell anemia AND mental disorders,” “sleep,” “substance-related disorders,” “pain,” “family relations,” “body dissatisfaction,” “interpersonal relations,” “psychology,” “pain AND psychology,” “medical psychology,” “social stigma OR social support OR quality of life OR sleep OR substance-related disorders OR Family relations OR body dissatisfaction OR interpersonal relations OR (pain AND psychology),” “sickle cell anemia AND social stigma OR social support OR quality of life OR sleep OR substance-related disorders OR Family relations OR body dissatisfaction OR interpersonal relations OR (pain AND psychology),” “social stigma OR social support or psychological adaptation or substance-related disorders or family relations or interpersonal relations or (pain AND psychology),” “sickle cell anemia AND [social stigma OR social support or psychological adaptation or substance-related disorders or family relations or interpersonal relations or (pain AND psychology)]”	368
Reference review	Review of reference list of identified articles	+30
Total articles	Total articles acquired from a detailed literature search	398
Screening	Selection using inclusion criteria	
• Language	Published in the English language	140
• Year of publication	Published between 1989 and 2023	149
• Excluding redundant papers	Repetitive or similar articles	85
• Relevance	Articles Focused on psychosocial issues in Sickle Cell Anemia and included participants diagnosed with SCA of any age group	98
•** Peer-reviewed** Quantitative and qualitative studies, including cross-sectional, longitudinal, and intervention studies, were considered for inclusion.	Articles published in peer-reviewed journals or regulatory repositories	84
Final selection	Articles meeting all inclusion criteria	80

### 2.3. Data extraction

A data extraction form was developed to collect relevant information from the selected studies. The extracted data included study characteristics (authors, year of publication, study design), participant characteristics (sample size, age, gender), psychosocial issues examined (including depression, anxiety, stigma, QOL, body dissatisfaction, family and peer support), assessment measures used, and key findings related to psychosocial issues in SCA. All Authors were part of the data extraction. The thematic areas were deduced for most themes based on the original authors’ conclusions in the section on other psychosocial issues. All authors read the entire manuscript and decided on the key takeaway.

### 2.4. Data analysis

A narrative synthesis approach was employed to analyze the extracted data. The findings from the included studies were organized and categorized based on the specific psychosocial issues investigated. Themes and patterns across the studies were identified, and critical results were summarized to provide a comprehensive overview of the psychosocial challenges in SCA patients.

### 2.5. Ethical considerations

Ethical approval was not required as this study involved the review of existing literature. However, efforts were made to ensure the confidentiality and anonymity of the participants in the original studies by reporting findings in an aggregated and de-identified manner.

### 2.6. Limitations

Potential limitations of this review include the exclusion of non-English language articles, the reliance on published literature, which may introduce publication bias, and the possibility of missing relevant studies despite the comprehensive search strategy.

## 3. Results

Following this methodology, a nonsystematic and comprehensive evaluation of the psychosocial issues in SCA was conducted. A total of 80 research articles were finally selected for the review (Table 1). The review findings will contribute to a better understanding of the impact of SCA on individuals’ psychosocial well-being, inform healthcare practices, and highlight areas for further research and intervention development (Table 2).

**Table 2:** Table showing psychosocial challenges faced by SCA patients and recommendations.

Psychosocial issues	Recommendations
Poor body image and behavioral/emotional problems	Provide psychological support and counseling services. Encourage self-acceptance and positive body image through education and empowerment. Promote healthy coping strategies and stress management techniques.
Stigmatisation	Raise awareness about sickle cell anemia to reduce misconceptions and stereotypes. Implement anti-stigma campaigns in schools, workplaces, and healthcare settings. Train healthcare professionals to provide non-judgmental care and support. Foster a supportive and inclusive environment for individuals with sickle cell anemia.
Neurocognitive deficits	Conduct regular neurocognitive assessments to identify specific areas of difficulty. Provide tailored educational interventions and support services. Collaborate with schools to implement appropriate accommodations and Individualized Education Plans (IEPs). Offer cognitive rehabilitation programs to improve cognitive functioning.
Workplace discrimination	Advocate for equal employment opportunities and reasonable accommodations. Educate employers about the unique challenges faced by individuals with sickle cell anemia. Establish workplace policies that protect against discrimination. Provide job training and vocational support to enhance employability.
Reduced quality of life	Develop comprehensive multidisciplinary care teams to address medical, psychological, and social needs. Offer pain management strategies and access to appropriate healthcare services. Facilitate support groups and peer networks to promote social connections and emotional well-being. Enhance community resources and support systems for individuals with sickle cell anemia.

SCA = sickle cell anemia.

## 4. Discussion

### 4.1. Depression and anxiety in sickle cell anemia

Mental disorders, such as depression and anxiety, are alarmingly common among SCA individuals, profoundly affecting their overall well-being. These disorders have far-reaching consequences, including increased pain, heightened opioid use, poor treatment adherence, interference with professional duties and schooling, and disruptions in family dynamics.^[16,17]^ Depression, in particular, is most prevalent among patients with SCA,^[18]^ with studies estimating that globally, 21.6% to 44% of adult SCA patients experience depression.^[19]^ Similar rates have been observed in the United States,^[20]^ and Africa,^[17]^ with a local study in Nigeria finding that nearly 50% of participants experienced depressive states.^[11,21]^ The chronic nature of the disease, the severity of symptoms, and the presence of psychosocial stressors contribute to the high prevalence of depression in this population.^[22]^ Moreover, depression and anxiety have been shown to predict worse mental and physical health outcomes in SCA patients.^[17,23–25]^ A cohort study conducted in Holland revealed that patients with anxiety and depressive disorders experienced severe and disabling chronic pain compared to those without these disorders.^[17,23–25]^ These mental disorders significantly impact overall health-related QOL more than the genotype.^[20]^ Recognizing the detrimental effects of mental disorders on individuals with SCA is crucial for providing comprehensive care. By addressing these mental health challenges, healthcare professionals can enhance the overall management and outcomes for individuals living with SCA.

### 4.2. Pain among patients with sickle cell anemia

Pain is a prominent feature of SCA that can profoundly impact the lives of individuals with the condition. Unlike typical pain experienced in other conditions, the pain in SCA is characterized by its unpredictable nature and the recurrent episodes of acute pain called VOC.^[26]^ During VOC, individuals with SCA experience varying degrees of neuropathic pain, including hyperalgesia (increased sensitivity to pain) and allodynia (pain from non-painful stimuli).^[27]^ Unfortunately, the treatment options for chronic pain in SCA are limited, with opioids being the primary choice.^[28]^ However, the use of opioids comes with its complications, including constipation, mast cell activation, addiction, and respiratory depression.^[28]^ Additionally, individuals with SCA often require higher doses of opioids compared to those with other acute or chronic diseases, making pain management more challenging.^[26]^ The limited efficacy of treating neuropathic chronic pain in SCA may be attributed to the diverse underlying pathophysiology that activates nociceptive fibers.^[29]^ This includes vascular dysfunction, inflammation, ischemia/reperfusion injury, and oxidative stress.^[29]^ Pain in SCA can be lifelong, influencing cognitive function and contributing to psychological distress.^[30]^ Pain in SCA can be lifelong, influencing cognitive function and contributing to psychological distress.^[28]^ Pain also directly affects emotional expression, behavior, and mood.^[29,30]^ Psychological factors, such as catastrophizing, play a role in pain modulation.^[31,32]^ Catastrophizing refers to an exaggerated negative appraisal of pain, and it can significantly impact pain perception during anticipated or actual pain episodes.^[33–35]^ It involves elements of rumination, magnification, and helplessness.^[35]^ Catastrophizing behavior is positively correlated with the degree of clinical pain.^[36]^ In children, higher levels of catastrophizing are associated with an increased risk of disability.^[37]^ Moreover, higher levels of catastrophizing have been linked to more significant depression and poorer QOL in adults with SCA.^[34]^ Understanding the complex interplay between pain, psychological factors, and SCA is crucial for effective pain management and improving the overall well-being of individuals with the condition.

### 4.3. Substance abuse among patients with sickle cell anemia

Chronic pain is a prevalent and debilitating symptom experienced by individuals with SCA. To manage this pain, pain medications, including opioids, are commonly used. However, the addictive properties of these medications can lead to further complications in SCA patients. The severity of pain in SCA can be inferred from the increased use of opioids and the incidence of abuse and addiction among SCA patients.^[38]^ Analgesics that are commonly abused among individuals with SCA include non-steroidal anti-inflammatory drugs, codeine, oxycodone, and more potent opioids like morphine, levorphanol, methadone, pentazocine, oxymorphone, and fentanyl.^[38–40]^ These medications, while effective in managing pain, can have addictive properties.

A comparative study in Nigeria reported a high incidence of pentazocine addiction among SCA patients who used it for VOC.^[41]^ In this study, 75% of SCA patients using pentazocine for VOC developed addiction, compared to 15% using other analgesics.^[41]^ Signs of pentazocine dependence, such as intense drug craving, excessive sweating, non-bone body pains, needle marks, excessive spending, begging, stealing, and poor academic performance, were observed.^[41]^ These findings highlight the severity of pain experienced by individuals with SCA and the need for alternative pain management strategies to minimize the risks associated with opioid use. It is essential to exercise caution when using pentazocine in SCA patients to prevent addiction and its detrimental consequences.^[41]^

Exploring non-opioid pain management options and developing tailored strategies for pain control in SCA are crucial. By identifying alternative approaches and implementing comprehensive pain management plans, healthcare providers can mitigate the risks of opioid addiction while effectively addressing the chronic pain experienced by individuals with SCA. This will ultimately enhance the overall QOL for SCA patients.

### 4.4. Sickle cell disease and sleep

Sleep is vital to overall well-being, particularly in children and teenagers who require proper rest for their growth and development.^[42]^ However, various factors can contribute to sleep disturbances, including stress from home and school.^[43]^ Research has shown that sleep problems are associated with increased physical, mental, and environmental health issues.^[43]^ In the case of individuals with SCA, episodes of acute pain known as VOC and sleep-disordered breathing have been identified as significant contributors to poor sleep.^[44]^ Children with SCA have a higher prevalence of sleep-disordered breathing than those without the disease. Studies have reported an increased incidence of obstructive sleep apnea syndrome in children with SCA, surpassing even the prevalence in the general pediatric population.^[44]^

Sleep-disordered breathing in SCA can lead to behavioral problems, learning difficulties, elevated blood pressure, bed-wetting, and reduced growth.^[44]^ These findings are consistent with another study that found a high prevalence of snoring and sleep-disordered breathing in children with SCA aged 2–14.^[45]^ Interestingly, a survey of Saudi children in the same age group showed even higher prevalence rates of obstructive sleep apnea, snoring, and bed-wetting compared to other countries.^[46]^ Additionally, there is a significant association between sleep-disordered breathing and sleepwalking in children and adults with SCA.^[47]^ Adults with SCA also experience a higher prevalence of sleep disorders, and there is an inverse relationship between pain and sleep quality in these patients.^[22,48]^

Understanding the impact of sleep disturbances in individuals with SCA is crucial for their overall well-being. Sleep-disordered breathing and its associated consequences can profoundly affect cognitive function, behavior, and physical health. Healthcare providers should be aware of these issues and consider incorporating sleep assessments and interventions into the comprehensive care of individuals with SCA. By addressing sleep disturbances, healthcare professionals can potentially improve the QOL and overall health outcomes for individuals living with SCA.

### 4.5. Interpersonal relationship between individuals with sickle cell anemia and family

Family dynamics play a critical role in supporting adolescents with SCD, providing essential comfort, motivation, and overall support that facilitate effective coping mechanisms and positive relationships with family members and peers.^[49,50]^ However, the challenges associated with SCA can significantly impact families, particularly in tropical countries like Nigeria, where the disease burden is substantial.^[51,52]^

Economic and psychosocial burdens have been identified as significant stressors in these families, including the inability to meet basic needs, loss of income due to caregiving responsibilities, financial strain related to SCA management, disruption of family interactions, increased conflicts, and neglect of other family members.^[51,52]^

Partners of adult patients also face unique challenges, such as frequent crises or hospitalizations, psychological stress, financial strain, social disruption, and stigmatization.^[53–56]^ Primary caregivers for children and adolescents with SCA often have limited time for socializing within the family, leading to anxiety and frustration for caregivers and patients.^[57]^ In this context, mothers primarily serve as caregivers and advocates for their children with SCA.^[58]^ While familial support is crucial, it can generate problems and tensions. Some parents may attempt to shield their children from social realities, leading to conflicts with adolescents who desire independence and autonomy.^[58,59]^ Adolescent patients may also experience feelings of guilt as they are aware of the physical and economic impact of SCA on their families.^[59]^ Knowledge about SCA has been found to influence psychological functioning and parent-child dynamics within families positively.^[49,60]^ Families with a comprehensive understanding of the disease tend to have better interpersonal relationships and overall adjustment. Effective coping strategies play a significant role in preventing families from becoming overwhelmed.^[61]^ Factors such as social support, socioeconomic status, family attitudes, the personality and developmental stage of the child, and other variables can influence coping mechanisms.

Public health education focused on the nature of the disease can enhance coping, improve interpersonal relationships, and positively impact families and society.^[61,62]^ Integrating psycho-educational interventions and psychosocial programs into comprehensive clinical management can provide vital support for SCA patients and their families.^[63]^ This approach can have reciprocal effects, improving coping in spouses and positively influencing the well-being of SCA patients.^[63]^

Healthcare professionals can improve the well-being of individuals with SCA and their families by understanding and addressing family dynamics. Further research and evidence-based interventions are needed to alleviate burdens and promote positive outcomes.

### 4.6. Interpersonal relationship between individuals with sickle cell anemia and peers

The knowledge and understanding of peers regarding SCA significantly influence the relationships with SCA individuals.^[64]^ This understanding plays a pivotal role in determining the support and acceptance received by individuals with SCA.^[64]^ Interestingly, patients with fewer hospitalizations tend to have more positive peer relationships.^[64]^ Conversely, frequent hospitalizations can lead to a reluctance to form connections, resulting in feelings of isolation and potentially triggering mood disorders.^[62]^ Regrettably, individuals with SCA often face an elevated risk of bullying and problematic peer relationships, including verbal and physical abuse.^[50,65,66]^ This risk is particularly pronounced for males with SCA, who may encounter more aggressive behavior from their non-affected peers.^[50]^ They may become targets due to their smaller size or be unfairly labeled as lazy when experiencing fatigue. These challenging experiences frequently contribute to social isolation and limited interactions with peers.^[50]^ As a protective meil puttychanism, individuals with SCA may adapt their behavior to avoid confrontation and potential abuse.^[50]^

Promoting understanding among peers is crucial to improving the experiences of individuals with SCA. Increasing awareness and empathy can foster positive relationships and reduce the risk of bullying. Providing support and resources can also contribute to their overall well-being. Further research is needed to develop effective strategies for addressing these issues and promoting positive peer relationships.

### 4.7. Other psychosocial issues

SCA can significantly impact a person self-image, including how they perceive their body and personality.^[67]^ This can cause poor body image and emotional problems, which can affect academic performance and achievement in children and adolescents with SCA.^[65,66,68]^

Studies have shown that SCA patients experience higher levels of body dissatisfaction, which correlates with increased stress, interpersonal distrust, and feelings of ineffectiveness.^[69,70]^ Stigmatization is also a common experience for individuals with SCA. In England, 75% of respondents reported stigmatizing incidents, such as being labeled lazy when experiencing fatigue.^[71,72]^ Similar findings were observed in a study conducted in South-West Nigeria, where 70% of subjects reported moderate to high perceived stigma.^[15]^ Stigmatization can arise from various factors, including using opioids for pain relief, race (being Black), delayed growth/puberty, socioeconomic status, and disease severity.^[71]^ It can come from unexpected sources such as health institutions, healthcare professionals, family, friends, and society.^[71]^ The experience of stigma in SCA has been associated with impaired sexuality, higher levels of perceived stress and pain, maladaptive coping, more emergency room visits, poor treatment adherence, and depressive symptoms.^[73–77]^

Functional impairment is another complication in managing SCA. Neurocognitive deficits have been observed in children and adults with SCA, such as lower intelligence quotient, visuomotor and executive dysfunction, poor working memory, attention and planning difficulties, slower processing speed, language impairments, and deficits in prosodic cues.^[78–81]^ Factors contributing to these deficits include stroke or silent infarcts, anemia severity, malnutrition, cerebral ischemia, and psychosocial factors such as low socioeconomic status, frequent hospitalizations, and family stress.^[79,81]^

Workplace discrimination is a common experience for SCA patients, with more than half of adult patients being unemployed.^[82,83]^ Siblings of SCA patients without the disease have a significantly higher employability rate than those with the disorder.^[82,84]^ Frequent hospitalizations and educational disruptions can lead to academic under-attainment and job under-qualification.^[83,84]^ For those who secure employment, periodic crises and absenteeism can impede job performance and security, and some SCA patients even report being fired due to discrimination by employers.^[82,83,85]^

Overall, SCA patients generally have a lower QOL, with medical complications, stigmatization, and frequent hospitalizations negatively impacting their health-related QOL.^[15]^ Studies consistently show poorer QOL in SCA patients than the general population, with significant impairments in physical functioning, emotional roles, social functioning, bodily pain, vitality, and public health perception.^[15,86,87]^ To improve the QOL for those with SCA, we need targeted interventions that combat stigma, promote positive self-image, enhance cognitive functioning, and facilitate inclusive and supportive workplaces. Further research is required to develop effective policies and interventions to address the multifaceted impact of SCA on individuals’ lives.

## 5. Conclusion

SCA is a significant burden, particularly among the black population. Efforts to increase awareness and promote premarital screening are ongoing. However, comprehensive management plans that address the psychosocial challenges associated with the disease are needed. This paper highlights the prevalence of depression, anxiety, substance abuse, sleep disturbances, body dissatisfaction, and peer bullying among individuals with SCA. These challenges, including painful crises, stigma, and economic difficulties, significantly impact treatment response, healthcare costs, and overall QOL. Targeted interventions and holistic care are necessary to address the mental health impact and improve well-being (Table 2). By recognizing and addressing the psychosocial burden of SCA, we can enhance the QOL and provide comprehensive support. This involves implementing interventions for mental well-being, coping strategies, and addressing unique disease challenges. Holistic care can alleviate psychosocial difficulties and improve the QOL for SCA patients.

## 6. Recommendation

Comprehensive and holistic care for individuals with SCA includes early identification and intervention, comprehensive support programs, patient and family education, enhanced pain management, integration of mental health into clinical care, school-based support, research and advocacy, community support groups, and continuous improvement (Table 2). Routine mental health screening should be implemented as part of standard care to identify psychosocial issues early. Comprehensive support programs should be integrated into clinical care, including mental health counseling, peer support groups, and educational workshops on coping strategies, pain management, and disease understanding. A multidimensional approach to pain management should be taken, utilizing non-opioid analgesics, physical therapies, and cognitive-behavioral interventions. Collaboration with schools is necessary to support students with SCA, implementing accommodations and fostering an inclusive school environment. Government investment in research is crucial for understanding the psychosocial burden of SCA and identifying effective interventions while advocating for increased funding and resources. Community engagement efforts should focus on reducing stigma and improving support networks, including establishing community-based support groups. Continuous assessment and evaluation and patient and family feedback will improve support services.

## Author contributions

**Conceptualization:** Emmanuel Aniekan Essien, Faithful Miebaka Daniel.

**Data curation:** Emmanuel Aniekan Essien, Blessing F. Winter-Eteng, Chinyere Uchechi Onukogu, Dominic Dennis Nkangha, Faithful Miebaka Daniel.

**Methodology:** Emmanuel Aniekan Essien, Faithful Miebaka Daniel.

**Project administration:** Emmanuel Aniekan Essien, Faithful Miebaka Daniel.

**Resources:** Emmanuel Aniekan Essien, Blessing F. Winter-Eteng, Faithful Miebaka Daniel.

**Supervision:** Emmanuel Aniekan Essien, Faithful Miebaka Daniel.

**Validation:** Emmanuel Aniekan Essien, Faithful Miebaka Daniel.

**Visualization:** Faithful Miebaka Daniel.

**Writing – original draft:** Emmanuel Aniekan Essien, Blessing F. Winter-Eteng, Chinyere Uchechi Onukogu, Dominic Dennis Nkangha, Faithful Miebaka Daniel.

**Writing – review & editing:** Emmanuel Aniekan Essien, Blessing F. Winter-Eteng, Chinyere Uchechi Onukogu, Dominic Dennis Nkangha, Faithful Miebaka Daniel.
